# In conversation with…John Burnard West

**DOI:** 10.1186/s13728-015-0029-6

**Published:** 2015-07-02

**Authors:** Ned Gilbert-Kawai

**Affiliations:** UCL Centre for Altitude, Space and Extreme Environment Medicine, Institute of Sport and Exercise Health, 170 Tottenham Court Road, London, W1T 7HA UK

*Whilst attending a conference last year, I was fortunate enough to be seated at lunch next to a very eminent octogenarian academic. Conversation ensued, and I was instantly captivated by his stories. These were not narratives filled with scientific facts, but rather anecdotes of his life and the pathways he had taken to get where he was today. Fascinated by such accounts, I set about the task of interviewing persons of scientific acclaim, to learn more about their life stories and unwritten tales. ‘In conversation with…’ therefore offers readers a chance to share an abridged version of these conversations.*

*(In view of the need to condense two and a half hours of conversation, the original interview text has been abridged, and then reviewed, revised, and approved by JBW).*

**At the interview for the Silver Hut expedition, Sir Edmund Hillary asked, ‘So, how high have you climbed?’**

**‘Well, I have never been on a mountain’ , I replied.**

**‘Oh’ , he said, ‘well that’s a bit of a setback. Why don’t you climb that flight of stairs?’**

**As I duly did so, Ed watched and said, ‘That’s ok, you’re in!’**

## Q. You were born in Adelaide in 1928. What did you parents do?

My father was an orthopaedic surgeon and my mother was a nurse.

## Q. Did their medical background influence your choice of career?

I do not know. I had to make a choice when I was about seventeen years old, and that is ridiculously young to decide what you want to do. Anyway, as the school exams had gone well, I was offered a bursary to attend university. Well, it was not difficult to decide where to go, as there only was one option in South Australia at the time—The University of Adelaide. Back then, as is still the case, I was interested in high-energy physics; however, for some reason I decided on medicine. Looking back, I am glad I chose medicine because it is a wonderful thing to do. You can always say at the end of your life that you probably have done more good than bad, which may not be the case for some other jobs.

## Q. Following graduation from university in 1959, you almost immediately moved to London. What prompted this?

I moved over after completing my year of residency. I had always been a great anglophile, and I wanted to experience London. Upon arriving, I only had one medical contact in London—a friend of a cousin of mine called Richard Bayliss. He worked in Hammersmith Hospital and at one time was a physician to the Queen. Through him I ended up meeting Charles Fletcher, and this was a critical step in my career for it was he who suggested that with my interest in physics, I could either go into cardiology or pulmonology. Well, as they happened to be starting a new pulmonology unit at Hammersmith Hospital, I chose pulmonology. I then spent one of the best years of my life at the MRC Pneumoconiosis Research Unit just outside of Cardiff learning the basics of pulmonary physiology. Following that, I returned to the Hammersmith Hospital, became a member of the Respiratory Research Group, and undertook a PhD with Philip Hugh Jones—initially looking at alveolar expired gas in patients with lung disease using a mass spectrometer. In fact, the first paper I wrote was on ventilation perfusion inequality as measured using expired gas analysis.

Much more exciting however, was that a year or so later, the Medical Research Council’s Cyclotron came on line at Hammersmith and became available for medical research. I can still remember a meeting with John McMichael, the chairman of medicine, who said ‘Here we have radioactive oxygen with half-life of two minutes being produced by the Cyclotron. Can anyone think of anything to do with it?’ ‘Well,’ I said, ‘why don’t we inhale it?’ And that’s just what we did. We first looked at COPD patients, to study the areas that were under-perfused. We then looked at normal subjects, and we were astonished to find that there was very little blood flow to the top of the upright human lung in seated normal subjects, and large amounts of blood flow in the bottom. This was the first direct demonstration of uneven blood flow occurring in the human lung and it really made our names.

## Q. During these early years at Hammersmith Hospital, you ended up on the ‘Himalayan Scientific and Mountaineering Expedition’ (The Silver Hut)

In the spring of 1960, I was sitting in a Physiological Society meeting at University College London, when the woman sitting beside me nudged me and asked if I knew that Griff Pugh was arranging a physiological expedition to Mount Everest. To be precise, Hillary was organising the expedition and Griff was the scientific director for it. Well anyway, it sounded interesting so I applied for it and was then summoned to London for an interview. There I met Sir Edmund Hillary who asked ‘So, how high have you climbed?’

‘Well, I have never been on a mountain,’ I replied.

‘Oh,’ he said, ‘well that’s a bit of a setback. Why don’t you climb that flight of stairs?’

As I duly did so, Ed watched and said, ‘That’s ok, you’re in!’

At the time I had been doing some work on pulmonary diffusing capacity. I could see that it could be measured, had done some modelling, and clearly the diffusing capacity of the lung could be a limiting factor at very high altitude. In retrospect, it was a good call for we demonstrated that at 19,000 feet, as you increase the work load, the arterial oxygen saturation plummets—so diffusion limitation of oxygen transfer is easily shown at that altitude…and in fact once you get near the summit it becomes a very critical thing. One of the most fascinating things to me is that the highest point in the world is extremely close to the limit of human tolerance—now that is a cosmic coincidence.

Well the Silver Hut expedition was a life-changing experience. It was very productive and since then, I have kept an interest in high altitude medicine and physiology.

## Q. On your return to Hammersmith, you spent a year with Rahn.

Yes, I spent a year with Hermann Rahn at the University of Buffalo in New York State. Rahn was a member of that extraordinary group of three people: Fenn, Rahn, and Otis. Interestingly, whilst none of them had a background in respiratory physiology, the bombing of Pearl Harbour in World War II had prompted them to focus their attention on respiratory physiology because of its relevance to pressure breathing in aircraft. They believed that if you could fly at a higher altitude, you could have an advantage in warfare. So because of the exigencies of war, they worked on respiratory physiology and laid the foundations of pulmonary gas exchange and pulmonary mechanics—an extraordinary story.

## Q. Whilst at Hammersmith Hospital, you also took a year of sabbatical leave in 1967–1968 at the National Aeronautics and Space Administration (NASA) Ames Research Center in California.

Well, in the mid-1960s, I had done a lot of work on the effects of gravity on the lung. It was natural to wonder what would happen in the absence of gravity during spaceflight. I knew people were working on the space programme—and of course Armstrong landed on the moon in July of ’69. I therefore wrote to an Englishman in NASA called John Billingham, and asked if it would be possible to spend a year in the NASA programme. I thought I might be able to get my foot in the door! I was subsequently offered a one-year fellowship, so I moved to Palo Alto—a lovely place in California—with my new wife Penelope. The year was interesting, but perhaps a little disappointing in that the NASA work there was not involved with the astronauts, but rather had more to do with animals. Whilst there however, John Billingham suggested that I put in a proposal to NASA to measure pulmonary function in humans in space. Well, I thought this to be a pretty outrageous proposal, because at that time there was no thought of doing any science in space. NASA was trying to think of a way of putting someone on the moon and getting him back, so I thought they would not be the least bit interested in the effects of gravity on the lung. Anyway, after some persuasion, I did submit the proposal and it was funded…and the funding continued for the next 30 years!

## Q. Following your time at NASA, I believe you decided to stay in America, and started work at a new medical school at the University of California, San Diego.

Yes, a move that was a bit unpopular with my wife at first. However, her mother was American, so it all worked out in the end! I was at a lung meeting in Aspen, and I remember vividly one morning over coffee an eminent pathologist from Yale approached me and told me about a new medical school being formed in San Diego. Well, I had hardly heard of San Diego—it was not on the map for most people interested in medical research, but they were looking for someone to act as an interface between medicine and physiology. The reason was that in this new medical school, they were going to teach physiology out of the department of medicine. Well, I had done a lot of clinical medicine at Hammersmith, and the emphasis at Adelaide had been very strong on clinical medicine, so they offered me a job. I agreed to join them and thus became one of the original faculty of the medical school. That was way back in 1969, and we have been there for 46 years—a wonderful period. The job started off particularly well, for the first NASA research-funding cheque of $100,000 arrived on my desk on the day I arrived—April 1st 1969! The funding allowed us to look at pulmonary function in space where it is unaffected by gravity—a potential problem for many systems in the astronauts. We mounted a big programme, and as there was not a vehicle that could produce zero gravity initially, we did some parabolic profile flights using Lear Jets. Then in the 1990s, Spacelab became available and we carried out an extensive series of studies.

## Q. In 1981, you led the American Medical Research Expedition to Everest (AMREE). What prompted this?

Well for some time I had been thinking that it would be fascinating to get measurements on the summit of Mount Everest. It is a nice end point because it is as far as you can go. We already had data showing that if you plot maximum oxygen consumption against barometric pressure, it decreases rapidly as barometric pressure falls, and it looked as though on the summit you would be right at the limit of human tolerance. When we were thinking about this in 1978, Everest was summited for the first time without supplementary oxygen by Messner and Habeler. Well this was a huge shock to a large number of people, including us. If no one had been able to reach the summit without supplementary oxygen, that would not have surprised us, but they had managed it.

In the early planning of the expedition we tried to graft a scientific team onto a regular climbing expedition. The objectives of the two groups are so different however, that this proved impossible. We therefore decided to put together a special expedition with physiological research as its primary objective—an expedition devoted to it. The most difficult part was raising the money. When you go along to someone and say that you want to climb Mount Everest, they say ‘Oh, what fun!’ and then you have to try and persuade them that it is a serious scientific project. The NIH initially voted against it because they said ‘it was too dangerous’, and ‘What are you doing using tax payers money to fund something as dangerous as this?’ The expedition was, however, eventually funded, and we even got a supportive letter from President Reagan.

The expedition team was a mix of six people whose main role was to climb, six so-called climbing scientists, and about six physiologists. Whilst I thought the mix could be problematic, I was astonished by the interest in the physiology of high altitude shown by the climbers. At one point we had terrible weather at base camp and we could not get into the icefall. To fill in the time, the non-scientists gave a couple of talks on things like avalanches and properties of snow, and the scientists gave a number of talks on the medical aspects. The climbers were fascinated by it. Anyway, as you know, the expedition was very fruitful and we successfully took measurements on the summit. It was a lot of hard work, and we also had some luck. Two extraordinary incidents occurred on the mountain. Prior to summiting, Chris Pizzo’s ice axe had been buried in an earlier storm at Camp V. He therefore set off up the mountain using just a tent pole. Fortunately however, before an ice axe became crucial, he just happened to find one left by a previous expedition lying in the snow! Second, and rather fortunately for him, as Peter Hackett was descending from the summit, he slipped on the Hillary step and fell headfirst. Remarkably as he fell, a boot got caught in a rock, halting his fall but leaving him hanging upside down!

## Q. In terms of the science, the achievements were absolutely remarkable and the breadth of your undertakings is astonishing. Exercise measurements to thyroid function tests, sleep studies to barometric pressure readings. Could you however sum up what you thought was the most important science from AMREE?

I think the most important thing was that we elucidated the physiological changes that make it possible to reach the summit of Mount Everest without oxygen. Interestingly, it seems that the most important of these is hyperventilation. Incidentally, after 34 years, we are still waiting for someone to repeat the measurements and tell us whether we were right!

## Q. Since those days, you have continued to do vast amount of research, and I read you have published over 400 papers. Included in this work is research related to the effects of gravity on the lung and stress failure in pulmonary capillaries. What now interests you in the world of research and how are you keeping busy?

So what do I do now? Well I edit this Journal, *High Altitude Medicine and Biology* which is very interesting. I am still doing some research. Pilots of high performance fighter aircraft develop brief periods of confusion and why this occurs is not known. There is a remarkable disconnect in that everything is known about the aircraft they fly, and essentially nothing about the pilot! So I am looking into that. Additionally, I writing a book called *Essays on the History of Respiratory Physiology*, which consists of bite-sized articles on people like Priestly, Lavoisier, and Humphry Davy. In fact, this has just been published by the American Physiological Society.

## Q. Away from research, did you continue clinical medicine throughout your career?

I did initially do some clinical work when I came to San Diego, but I was not hired for that, but rather to run the physiology course. I have, however, kept up with pulmonary medicine, and I often talk to people in critical care as they are interested in the measurements we made because it relates to very severe hypoxia which is relevant to them.

## Q. Did running the physiology course lead to your keen interest in education?

Yes indeed. I ran the physiology course for about 30 years. However, when I initially started teaching at UCSD, it was to first year medical students and I had never taught them before. Hammersmith Hospital had all been postgraduate students. Once I started teaching, I found it both challenging and interesting, and I realised there were no appropriate texts on respiratory physiology. I therefore very quickly put together some lecture notes, and it was about three years later when I produced the red book: *Respiratory Physiology—the essentials*. Now that is in its ninth edition, and I have also recorded the contents as YouTube lectures which are popular. Funnily enough, I now get emails about the videos from countries I have never heard of!

## Q. Additionally you co-wrote the *High Altitude Medicine and Physiology* book—again another monumental text that is still going strong.

That all started with Mike Ward when he wrote a book called *Mountain Medicine*. He was a surgeon, not particularly interested in physiology, but had a working knowledge of altitude medicine from the 1953 expedition. I believe he and Jim Milledge then got together and wanted to extend it so they asked me to join them. The book is now in its fifth edition, and has changed appreciably over the editions. The original one had a lot of stuff from Mike Ward—pictures of people with enlarged thyroids, more of a sort of bits and pieces text, but we have tried to make it a more organised book which I think it is now.

## Q. May I now ask you a few generic questions? I know you have had hundreds of publications, but do any stand out to you? Any prize publications?

Well, I have a couple that I am quite proud of like the one on pulmonary gas exchange on the summit of Mount Everest. And also the work we did in space—I have done one or two reviews of pulmonary function in space that set out most of what we have done. I also like the paper on stress failure in pulmonary capillaries, for that was the first time anyone had suggested that the capillaries were vulnerable to breaking and I think it is terribly important in all sorts of areas.

## Q. In your opinion, what kind of questions lie unanswered?

I think there is still a lot to do at high altitude. There are, for example, millions of people who live at high altitude and have not had the attention that they deserve because they are of low socio-economic status. Examples include the highlands of Peru. Another interest is with people working at high altitude. Mines are being placed at increasingly high altitudes—there is a new mine in Peru at 4900 m. They also now have telescopes at 5000 m. Additionally, the aviation problem I mentioned is terribly interesting and someone needs to sort that one out. So yes, I think there are still plenty of interesting issues that need addressing.

## Q. If I was to give you a fully funded lab and all the time in the world, are there any of those you would particularly like to study?

Sadly no one will give me any money now! But if they did then I am very interested in the avian lung at present. I believe that the avian lung is superior to the mammalian lung in many respects, and we have done a lot of work on it. One of the things that comes out is that the avian lung capillaries appear to be remarkably rigid unlike the case in the mammalian lung. In the mammalian lung, you get distension which is valuable as it allows the pulmonary artery to be kept in check when the cardiac output increases. The prediction in birds on exercise is that the pulmonary artery pressure will go up considerably, but no one has ever measured it. We were working on it, but funding is so difficult to come by now.

## Q. Do you have any particularly fond memories of the research over the years?

I have been terribly lucky right from the very beginning. If I had not got into Hammersmith, I could not have started work with the mass spectrometer and developed the interest in gas exchange that I have kept all the time. I could not have done the Oxygen-15, work which turned out to be critical in terms of my career. And then the Silver Hut was a fortunate event—all that from a nudge from the person sitting next to me. And then Silver Hut led to AMREE. So you know, I have been very lucky indeed.

## Q. Are there any particular people over the years that have been of particular influence on you?

Well Hermann Rahn was a very important person in the gas exchange business, but I have been influenced by many, many people over the years. The pulmonary physiology community in the early days, and when I say early days I mean in the 60s and 70s, was a wonderful community. It was fairly close knit, and a very stimulating period, particularly as applied to clinical problems. Sadly that is kind of finished now and the discipline has become much more diversified. If I read the journals, I do not see a lot that interests me anymore in terms of pulmonary function. There are lots of people working on molecular things but this area still has to prove itself.

## Q. And finally, is there anyone in history that you would have liked to have sat down with and had a conversation with?

As I am very interested in the history of physiology, it would have been lovely to have sat down with Haldane or Barcroft. Now that would have been a fascinating conversation.

